# Therapy with Sodium Stibogluconate in Stearylamine-Bearing Liposomes Confers Cure against SSG-Resistant *Leishmania donovani* in BALB/c Mice

**DOI:** 10.1371/journal.pone.0017376

**Published:** 2011-03-10

**Authors:** Jayeeta Roychoudhury, Roma Sinha, Nahid Ali

**Affiliations:** Infectious Diseases and Immunology Division, Indian Institute of Chemical Biology, Jadavpur, Kolkata, India; Universidade Federal de Minas Gerais, Brazil

## Abstract

**Background:**

Resistance of *Leishmania donovani* to pentavalent antimonials, the first-line treatment of visceral leishmaniasis (VL), has become a critical issue worldwide. Second-line and new drugs are also not devoid of limitations. Suitable drug-delivery systems can improve the mode of administration and action of the existing antimonials, thus increasing their clinical life.

**Methodology/Principal Findings:**

We investigated the efficacy of sodium stibogluconate (SSG) in phosphatidylcholine (PC)–stearylamine-bearing liposomes (PC-SA-SSG), PC-cholesterol liposomes (PC-Chol-SSG) and free amphotericin B (AmB) against SSG-resistant *L. donovani* strains in 8-wk infected BALB/c mice. Animals were sacrificed and parasites in liver, spleen and bone marrow were estimated 4-wk post-treatment by microscopic examination of stamp smears and limiting dilution assay. A set of PC-SA-SSG and AmB treated mice were further studied for protection against reinfection. Serum antibodies and cytokine profiles of ex-vivo cultured splenocytes were determined by ELISA. Uptake of free and liposomal SSG in intracellular amastigotes was determined by atomic absorption spectroscopy. Rhodamine 123 and 5-carboxyfluorescein, known substrates of Pgp and MRP transporter proteins, respectively, were used in free and liposomal forms for efflux studies to estimate intracellular drug retention. Unlike free and PC-Chol-SSG, PC-SA-SSG was effective in curing mice infected with two differentially originated SSG-unresponsive parasite strains at significantly higher levels than AmB. Successful therapy correlated with complete suppression of disease-promoting IL-10 and TGF-β, upregulation of Th1 cytokines and expression of macrophage microbicidal NO. Cure due to elevated accumulation of SSG in intracellular parasites, irrespective of SSG-resistance, occurs as a result of increased drug retention and improved therapy when administered as PC-SA-SSG versus free SSG.

**Conclusions/Significance:**

The design of this single-dose combination therapy with PC-SA-SSG for VL, having reduced toxicity and long-term efficacy, irrespective of SSG-sensitivity may prove promising, not only to overcome SSG-resistance in *Leishmania*, but also for drugs with similar resistance-related problems in other diseases.

## Introduction

Leishmaniasis is a disease caused by different species of protozoa of the genus *Leishmania* that are transmitted by Phlebotomine sandflies. It has traditionally been classified in three different clinical forms, visceral (VL), cutaneous (CL) and mucocutaneous leishmaniasis (MCL), which have different immunopathologies and degrees of morbidity and mortality. VL is caused by *Leishmania donovani* in the Indian subcontinent, Asia, and Africa, *L. infantum* in the Mediterranean basin, and *L. chagasi* in South America and is usually fatal if left untreated [1], [2]. In the absence of vaccines, pentavalent antimonials like sodium stibogluconate (SSG) and meglumine antimoniate remain the first line therapy for CL and VL for over half a century, and are still in use in many parts of the world. The prolonged treatment requiring parenteral administration, toxicity, and the emergence of significant resistance are all factors limiting the drugs' usefulness [3]. Although few other options are available such as amphotericin B (AmB), miltefosine, paromomycin, and lipid-conjugated formulations of AmB, they also suffer from one or more limitations [4]–[7]. Approaches to overcome antimonial resistance include use of alternative drugs which as mentioned earlier, are not devoid of limitations, or combination therapy having synergistic effects to check the further development of resistance [8]–[12]. Additionally, effective therapies against SSG-resistant parasites include reversing their resistant nature with verapamil or buthionine sulfoximine in combination with SSG [13], [14]. Reports of resistance development towards newer drugs [15], considerable variation in resistance mechanisms in field isolates [16], and lack of progress in drug discovery add to the severity of the problem. A lot of research has focused on understanding the probable mechaninsms of drug resistance in *Leishmania* with main focus on SSG resistance. The reason for the emergence of resistance seems complex and multifactorial. As reviewed by Croft et al. [8], reduction of drug concentration within the parasite, either by decreasing drug uptake or by increasing efflux/sequestration of the drug, constitutes the primary mechanism of antimonial resistance; other potential resistance mechanisms include inhibition of drug activation, inactivation of active drug, and gene amplification. In the light of the above complications, improvement in the mode of administration and action of the existing and proven antileishmanials can become an important strategy in the management of VL [17].

Liposomes are artificially prepared vesicles made of lipid bilayer. They can act as targeted drug delivery agents to macrophages in parasitic infections [18]. Liposomal SSG formulations against SSG-responsive parasites have been shown to enhance the antileishmanial activity of free SSG [19]–[25]. Carter et al. reported a differential organ dependent activity of non-ionic vesicular formulation of SSG in some clinical isolates of *Leishmania* which did not respond to free SSG [26], but information on the development of long lasting protective cure against SSG-resistant parasite infection with liposomal SSG therapy is lacking. Earlier, we reported that cationic liposomes with egg phosphatidylcholine (PC) and stearylamine (SA) had leishmanicidal activity [27], [28] and entrapment of SSG (PC-SA-SSG) enhanced their potentiality against chronic VL in in vivo murine model [29]. PC-SA liposomes kill *Leishmania* by specific interaction with surface phosphatidylserine (PS) of promastigotes and amastigotes [30]. We had earlier reported on the successful immunomodulation of the host with a liposomal AmB formulation as a curative strategy in treating SSG-responsive VL infection [31]. Moreover, liposomes with their slow release property are known to circumvent the membrane efflux pathways thus increasing the intracellular retention of drug [32].

In the present study we compared the potential of single dose PC-SA-SSG therapy with PC-cholesterol formulation (PC-Chol-SSG) and AmB against SSG-resistant *L. donovani* GE1F8R and CK1R infection in BALB/c mice. These data add a new dimension to the therapy of SSG-unresponsive *L. donovani* infection. The insight into the probable mechanisms by which PC-SA associated drug works gives a broader perspective to this study in aiming other infections with similar immunological profiles and drug resistant phenotypes.

## Materials and Methods

### Animals and parasites

BALB/c mice and Golden Syrian hamsters, bred in the animal facility of Indian Institute of Chemical Biology, Kolkata, India, were used for the studies. The studies were approved by the Institute's Animal Ethical Committee (147/1999/CPCSEA) and animals were handled according to their guidelines. *L. donovani* SSG-sensitive AG83 (MHOM/IN/1983/AG83) and resistant, GE1F8R (cloned from MHOM/IN/1989/GE1) and CK1R (isolated as MHOM/IN/1995/CK from a SSG-unresponsive patient) strains were maintained in vivo by intracardiac injection into hamsters [33]. Transformation of amastigotes from infected hamster spleen to promastigotes was carried out at 22°C in medium-199 (Sigma-Aldrich, St. Louis, MO) supplemented with penicillin G sodium (l00 U/ml), streptomycin sulfate (100 mg/ml) and 10% heat inactivated fetal bovine serum (FBS) (Sigma-Aldrich) and subcultured in the same medium [31].

### Entrapment of SSG, rhodamine 123, and 5-carboxyfluorescein in liposomes

Liposomes were prepared with PC form egg yolk and Cholesterol (Sigma-Aldrich) or SA (Fluka, Buchs, Switzerland) at 7:2 molar ratios, respectively. For encapsulation of SSG (Gluconate Health Limited, Kolkata, India) the lipid film containing 20 mg of PC was mixed with either 2 mg of SA or 3 mg of Chol and dispersed in 0.02 M phosphate buffered saline (PBS) pH 7.4 containing 1 mg/ml of SSG and sonicated in an ultrasound probe sonicator (Misonix, Farmingdale, NY) for 2 min with intervals on ice. Unentrapped SSG was removed by centrifuging thrice at 60,000×g, for 30 min each [29]. The amount of SSG entrapped in PC-SA and PC-Chol liposomes as determined colorimetrically [29], ranged from 15 to 20 *µ*g/mg of PC and the efficiency of entrapment was approximately 30 to 40%. To entrap rhodamine 123 (Rh 123) (Calbiochem, San Diego, CA) in PC-SA (PC-SA-Rh 123), freshly prepared Rh 123 solution in chloroform was added to the lipids [34] and liposome was prepared as described above. For entrapment of 5-carboxyfluorescein (CF) (Sigma), lipid film was dispersed in PBS containing 50 mM CF [35] and liposomes were prepared as above. The entrapment efficiency estimated from a standard curve of the respective dyes after disrupting the liposomes with 1% TritonX-100 (Sigma) and measuring the fluorescence intensity with Fluorescence spectrophotometer (F-7000 FL spectrophotometer, Hitachi High Technologies, Japan) (505 nm/535 nm for Rh 123 and 492 nm/517 nm for CF) was found to be nearly 90% for Rh 123 containing liposome and 75% for CF containing liposome.

### In vitro antileishmanial assay and quantification of intracellular SSG of amastigotes

Peritoneal macrophages isolated from BALB/c mice were pooled and cultured at 37°C in 5% CO_2_ in RPMI-1640 (Sigma-Aldrich) supplemented with FBS and antibiotics as described above. SSG-sensitive AG83 and resistant GE1F8R promastigotes were allowed to infect peritoneal macrophages (10∶1) for 3 h. Infected cells were thereby treated with various doses of liposomal or free SSG, for 72 h at 37°C. The cells were then fixed and stained with Giemsa for microscopic determination of intracellular parasite numbers per 200 host cells [29]. In parallel sets of experiments, cells were scraped and amastigotes were freed from their host cells, as described [36]. Dried amastigotes were digested with nitric acid overnight and diluted with deionised water for antimony analysis by atomic absorption spectroscopy (AAS) (Perkin-Elmer 4100 ZL, CA, USA) [37].

### Dye uptake and retention assay

Rh 123 uptake and retention studies were performed according to Kang et al. with slight modifications [34]. *L. donovani* AG83 and GE1F8R promastigotes were washed and resuspended (2×10^5^ parasites/ml) in serum free M-199, and incubated along with different concentrations of free Rh 123 for indicated periods of time at 22°C. For retention studies, parasites were incubated with 250 ng/ml of free or entrapped Rh 123 for 4 h, washed and further incubated in media free of Rh 123. In case of CF, cells were preincubated with 2 µM of the dye [38]. At indicated time points, parasites were washed thrice in PBS and finally lysed in 0.1% Triton X-100. The intracellular dye concentrations were determined by measuring fluorescence intensity of the cell lysates and comparing them with the standard curves as above. The percent retention of free *vs* liposomal dyes were calculated with respect to controls.

### Infection of mice and treatment regimen

For experimental infections, BALB/c mice (4–6 weeks) were injected via the tail vein with 2.5×10^7^ hamster spleen-derived *L. donovani* amastigotes (in 200 µl 0.02 M PBS/mouse), and for reinfection, the same number of amastigotes were injected 12-wk after the primary infection. Eight weeks postinfection, groups of animals were treated intravenously with either 300 mg/kg of free SSG, or 12 mg/kg of SSG entrapped in PC-SA or PC-Chol liposomes. Amphotericin B deoxycholate (AmB) (kind gift from R. P. Goswami and B. Saha of School of Tropical Medicine, Kolkata, India) was administered intravenously at a single dose of 2 mg/kg/mice. Mice were sacrificed 4-wk post-treatment, and spleen and liver parasitic loads were determined from Giemsa-stained impression smears and reported as Leishman Donovan Units (LDU), calculated as the number of parasites per 1000 nucleated cells x organ weight (in mg) [31], [39]. In selected groups, a weighed piece of spleen or liver from experimental mice was first homogenized in Schneider's Drosophila medium (Invitrogen Corporation, Carlsbad, CA) supplemented with 10% FBS, and then diluted in the same medium to a final concentration of 1 mg/ml. Five-fold serial dilutions of the homogenized tissue suspensions were plated in 96-well plates and incubated at 22°C for 21days, with periodic checking at 7-day intervals for viable and motile promastigotes. The reciprocal of the highest dilution that was positive for parasites was considered to be the parasite concentration per milligram of tissue. The total organ parasite burden was calculated using the weight of the respective organs [31]. Infection in bone marrow was calculated as parasites/1000 host cell nuclei [31]. Mice reinfected at 4-wk post-treatment were sacrificed at 20-wk of initial infection and organ parasite burden was determined as above.

### Detection of IgG isotype levels in the serum

Mice were bled 4 wk after treatment, and sera were stored at -20°C until use. Antigen-specific serum immunoglobulin (Ig)-G isotype antibody response was measured by conventional ELISA as described [31]. Briefly, 96-well ELISA plates (Maxisorp, Nunc, Roskilde, Denmark) incubated overnight at 4°C with 2.5* µ*g/well *Leishmania* membrane antigen (LAg) [31], were blocked and further incubated with mice sera (1∶1000 dilutions) for 1 h, washed thoroughly, followed by 1 h incubation at 37°C with peroxidase-conjugated goat anti-mouse IgG1 or IgG2a antibodies (BD Pharmingen, San Diego, CA). The plates were then developed for color reaction with substrate solution (*o*-phenylenediamine dihydrochloride, 0.8 mg/ml in phosphate-citrate buffer (pH 5.0), containing 0.04% H_2_O_2_) for 30 min, and absorbance was measured on ELISA plate reader (Thermo Electron Corporation, Waltham, MA) at 450 nm.

### Splenocyte-proliferation and analysis of cytokines

Splenocyte-proliferation assay was performed for different experimental groups. Cell suspension was prepared by mechanical disruption of spleen, followed by red blood corpuscles (RBC) lysis with 0.14 M Tris buffered NH_4_Cl. After several washings in RPMI 1640 medium, cells were resuspended in complete medium (RPMI 1640 supplemented with 10% FBS, l00 U/ml penicillin G sodium, 100 mg/ml streptomycin sulfate and 50 mM β-mercaptoethanol [Sigma-Aldrich]). Viable mononuclear cell number was determined by counting Trypan blue unstained cells in a hemocytometer. Cells were plated in triplicate at 2×10^6^ cells/ml concentrations in 96-well plates (Nunc, Roskilde, Denmark) and allowed to proliferate for 72 h at 37°C in 5% CO_2_ incubator in presence of 12.5* µ*g/ml LAg [40]. Cells were pulsed further for 18 h with 0.5* µ*Ci of [^3^H] thymidine/well (Amersham Biosciences, Buckingham-shire, UK), harvested on glass fiber paper, and radioactivity was measured in a liquid scintillation counter (Beckman Instruments, Fullerton, CA). In parallel experiments, cytokine production by splenocytes was determined by ELISA kit (BD Biosciences, San Diego, CA), as per manufacturer's instruction. For in vitro depletion, total splenocytes were incubated with 1* µ*g/10^6^ cells of anti-CD4^+^ or anti-CD8^+^ mAbs (BD Biosciences) for 1 h at 4°C. Cells were washed and cultured in LAg as above. The efficacy of depletion was documented on each experiment by flow cytometry. Splenocytes from normal mice were cultured similarly in complete medium with different doses of free SSG or entrapped in PC-SA and PC-Chol liposomes for 48 h with or without LPS (2.5* µ*g/ml) [31] and cytokine production was determined as above.

### Quantification of Nitric Oxide

Nitric oxide (NO) content in the culture supernatants from LAg-pulsed splenocytes cultured for 72 h was analyzed by Griess assay method according to Ding et. al., using NaNO_2_ diluted in culture medium as standard [41]. Briefly, the mixture of Greiss reagent (1% sulfanilamide and 0.1% *N*-(1-naphthyl) ethylenediamine dihydrochloride in 2.5% H_3_PO_4_) and culture supernatant at 1∶1 ratio was incubated for 15 min at room temperature, and the OD was determined at 550 nm by ELISA reader (Thermo Electron Corporation, Waltham, MA).

### Statistical analysis

Statistical analyses were done using GraphPad Prism (GraphPad Software,v.5.0, San Diego, CA) software. A two-tailed Student's t-test was used to compare the significance between two groups. A one-way ANOVA (non-parametric) was used to compare more than two groups, followed by Tukey's multiple comparison test. Differences were considered statistically significant at p<0.05.

## Results

### Cure and protection against reinfection conferred by PC-SA-associated SSG against drug-resistant *L. donovani* infection in mice

**Figure 1:**
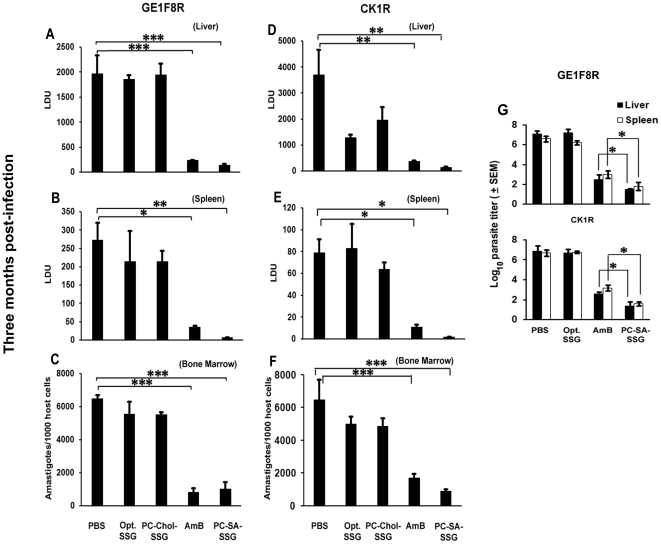
Parasite burden in treated BALB/c mice after *L. donovani* challenge infection. Parasite loads of liver, spleen, and bone marrow in murine model of established visceral leishmaniasis after treatment with 300 mg/kg of free sodium stibogluconate (SSG), 12 mg/kg of SSG entrapped in phosphatidylcholine-stearylamine (PC-SA) or phosphatidylcholine-cholesterol (PC-Chol) liposomes and 2 mg/kg amphotericin B (AmB). Mice were infected with *L. donovani* amastigotes of either GE1F8R or CK1R strains. At 8 week postinfection (p.i.) mice were treated with a single dose of various drugs by intravenous injection. Mice were sacrificed after 4 weeks of treatment for determination of (A, D) liver, (B, E) spleen, and (C, F) bone marrow parasite loads. Untreated, infected mice were used as controls. Liver and spleen parasite burden were determined by stamp-smear method and expressed as Leishman Donovan Units (LDU), and bone marrow parasite load in cell smear prepared from femur bone marrow and expressed as amastigotes/1000 bone marrow nuclei. Data represent mean ± SEM (n = 5 mice per group), representative of two similar experiments. * p<0.05; ** p<0.01; *** p<0.001. (G) Eight week infected mice received optimal SSG, PC-SA-SSG and AmB. Parasite burden determined 12 weeks following infection reflects the mean log_10_ parasite burden ± SEM determined by the limiting dilution assay (LDA) (n = 5 mice per group) with PBS treated group as control. Data are representative of two similar experiments. * p<0.05 compared to AmB therapy.

**Figure 2:**
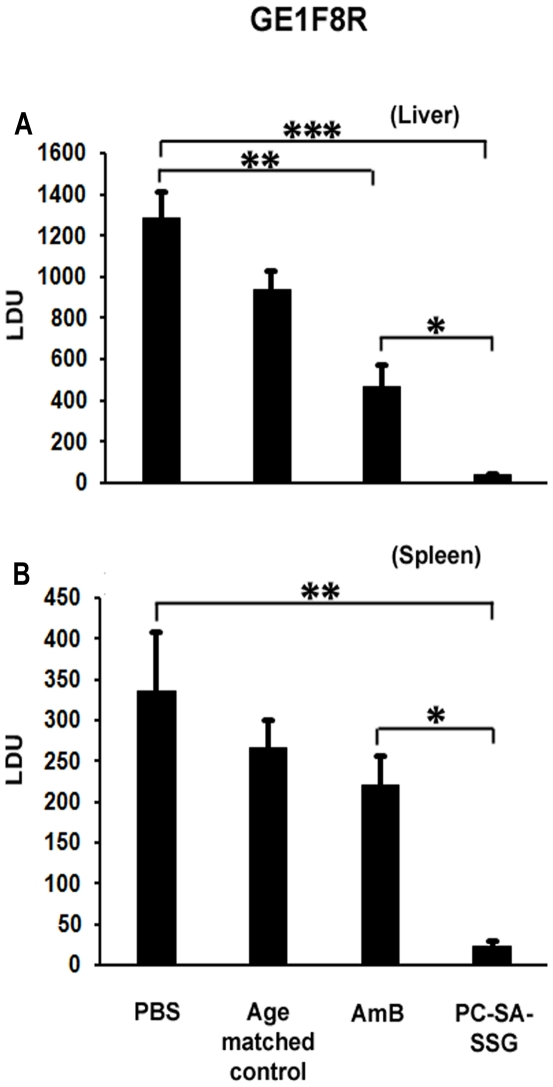
Parasite burden in cured BALB/c mice after reinfection with *L. donovani.* Cured mice (PC-SA-SSG and AmB treated) after 4 weeks of treatment along with naïve age-matched controls were reinfected with similar dose of virulent amastigotes and at 20 weeks of primary infection, were sacrificed and liver (A) and spleen (B) parasitic loads were determined by stamp-smear method and expressed as Leishman Donovan Units (LDU). Data represent mean ± SEM (n = 3-5 mice per group), representative of two similar experiments. * p<0.05; ** p<0.01; *** p<0.001.

Earlier we reported a profound synergistic activity of SSG entrapped in PC-SA liposome in both in vitro and in vivo models of SSG-non-resistant VL [29]. Here, therapeutic potency of PC-SA-SSG was compared with other formulations in susceptible BALB/c mice infected with virulent strains of SSG-resistant *L. donovani*, GE1F8R and CK1R, to validate the strain-independent efficacy of PC-SA-SSG. Progressively infected mice on treatment with free SSG and PC-Chol-SSG failed to suppress parasite load in GE1F8R, although they showed partial activity against CK1R strain (Figure 1). Interestingly, PC-SA-SSG suppressed liver parasitic load by 93% and 97%, respectively in GE1F8R and CK1R infected mice, which were comparable to AmB therapy (Figure 1A, D). In contrast to free SSG and PC-Chol-SSG, significant suppression with PC-SA-SSG was also achieved in spleen (98% and 96%) (Figure 1B, E) and bone marrow (84% and 86%) (Fig. 1C, F) of GE1F8R and CK1R infected mice respectively (p<0.05). Strikingly, the more sensitive limiting dilution assay demonstrated superiority of PC-SA-SSG over AmB in suppressing liver and splenic parasite burden (Figure 1G) in both GE1F8R and CK1R infected mice (p<0.05). To further strengthen our findings, 8-wk-infected BALB/c mice treated with PC-SA-SSG and AmB were re-infected with GE1F8R intravenously 4-wk after therapy. Age-matched normal mice were also infected simultaneously and were considered as age-matched controls. In comparison to AmB, mice treated with PC-SA-SSG were more resistant to re-infection with GE1F8R (p<0.05) (Figure 2A, B). We observed only a slight increase in liver and spleen parasite burden in the PC-SA-SSG-treated group till 20-wk of initial infection.

### In vitro antileishmanial activity and differential accumulation of SSG induced by PC-SA and PC-Chol liposomes

**Figure 3:**
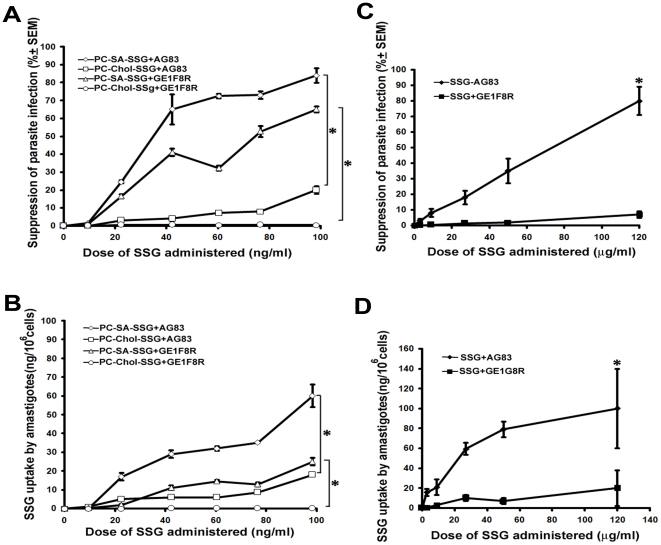
Parasite suppression in *L donovani*-infected macrophages and SSG accumulation in intramacrophagic amastigotes. Both AG83 and GE1F8R infected peritoneal macrophages were treated with (A, B) PC-SA-SSG, PC-Chol-SSG, and (C, D) free SSG for 72 h. (A, B) The treatment-induced percentage suppression of parasites was calculated in comparison to respective untreated-control. (B, D) Total SSG uptake by intramacrophagic amastigotes following various treatments was estimated by atomic absorption spectroscopy (AAS), and represented as nanogram (ng)/10^6^ cells. Data represent the mean ± SEM of three independent experiments. * p<0.05 compared to AG83 and GE1F8R strains treated with PC-Chol-SSG.

In accordance to our in vivo findings, PC-SA-SSG could induce significantly higher suppression towards parasite infection (p<0.05) compared to PC-Chol-SSG treatment in both SSG-sensitive AG83 and SSG-resistant GE1F8R infected macrophages (Figure 3A). SSG resistance in *Leishmania* involves reduction in intracellular SSG accumulation, either as a result of its reduced influx or increased efflux from the parasite and/or macrophages [4]. The suppression in parasite infection as observed in our experiments was associated with an elevated accumulation of SSG in the intracellular amastigotes of both AG83 and GE1F8R (p<0.05) parasites compared to PC-Chol-SSG as measured by AAS (Figure 3B). Free SSG on the other hand showed negligible antileishmanial activity, accompanying poor drug accumulation within resistant GE1F8R, compared to sensitive AG83 (Figure 3C, D) (p<0.05).

### PC-SA incorporation increases retention of rhodamine 123 and 5-carboxyfluorescein in *Leishmania*

**Figure 4:**
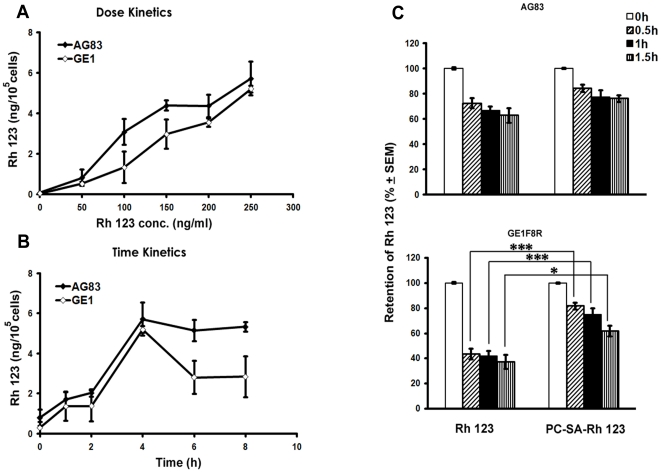
Intracellular retention of free and liposomal rhodamine 123 in *L.donovani* promastigotes. Rhodamine 123 (Rh123) uptake and retention study was performed in AG83 and GE1F8R strains with Rh123. Parasites were incubated with (A) different concentrations of free Rh123 for 4 h and (B) for different time periods. At indicated time points parasites were washed, lysed and fluorescence intensity of the cell lysates measured. Data reperesent mean ± SEM of two independent experiments performed in triplicates and expressed as ng of Rh123/10^5^ cells. (C, D) In another set of experiments, parasites incubated for 4 h with 250 ng/ml of either free or liposomal Rh123 were washed and reincubated in medium free of Rh 123 for different times followed by lysis. The percent retention of Rh123 was calculated for each group compared to respective controls. Data represent mean ± SEM of three independent experiments each performed in duplicate. * p<0.05; *** p<0.001

We and others have demonstrated the increased efficacy of vesicular SSG in the treatment of VL [19]-[25], [26]. The exact mechanism by which this strategy works however remains to be studied. One of the mechanisms is the entrapment of drugs in liposomes, which results in circumvention of Pgp mediated drug efflux, and is well demonstrated in cancer therapy [34]. Drug resistance in *Leishmania* has been variously described and involves active efflux systems working in the parasite and/or macrophages [4]. Thus we speculated that entrapment of SSG in PC-SA can prevent its exposure to the active pumps in turn increasing its intracellular concentration. To investigate if PC-SA can circumvent the resistance mechanism active in the parasite, we used Rh 123 and CF, substrates for Pgp and MRP family respectively. Rh 123 concentrations of 250 ng/ml and uptake time of 4 h were chosen from the dose and time kinetic studies using free Rh 123 (Figure 4A, B). When AG83 and GE1F8R parasites, preincubated with either free Rh 123 or PC-SA-Rh 123, were again incubated in dye-free medium, the intracellular Rh 123 concentration decreased with time regardless of the parasite strain (Figure 4C). However, the fall was faster and greater in GE1F8R compared to AG83 for free Rh 123. Interestingly, loading Rh 123 into PC-SA liposome although showed no difference compared to free dye in AG83, it significantly increased the retention capacity of GE1F8R (1.6 to1.8 folds between 0.5 to 1.5 h) (p<0.05 to 0.001). Loading the liposomes with CF produced a similar profile in SSG-unresponsive parasites. When GE1F8R promastigotes preincubated with the free dye were further incubated in dye free medium, 65.5±1.55%, 57.27±1.714%, and 37.57±1.068% dye were retained after 0.5 h, 1 h and 1.5 h respectively. When liposomal CF was used the retention increased to 83.21±1.76%, 74.28±2.73%, and 67.23±1.28%, with a significant rise of 0.8 to 0.6 folds between 0.5 h and 1.5 h (p<0.01 to 0.001). Free Rh 123 was effluxed out more vigorously then free CF till 1 h of incubation after which CF levels dropped to 37%.

### Immunomodulatory effects of PC-SA-SSG on normal mice splenocytes

**Figure 5:**
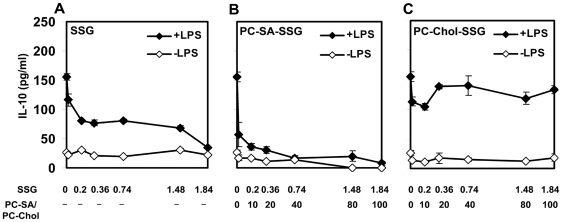
Immunomodulatory activity of free and vesicular SSG on splenocytes of normal BALB/c mice. Splenocytes of normal healthy mice were incubated with various concentrations of (A) free SSG, (B) PC-SA-SSG, and (C) PC-Chol-SSG with or without LPS (2.5 µg/ml) for 48 h at 37°C with 5% CO_2_. IL-10 was measured from culture supernatants by enzyme-linked immunosorbent assay (ELISA). Each symbol represents mean cytokine level ± SEM, representative of three independent experiments.

The protective chemotherapeutic response demonstrated by PC-SA-SSG prompted us to investigate its possible immunomodulatory role in vitro. As reported earlier [42], SSG could suppress the disease promoting IL-10 production in normal LPS-pulsed splenocytes (Figure 5A). SSG (0.36 µg/ml) entrapped in PC-SA (20 µg/ml) further brought about a 1.7-fold higher reduction in IL-10 compared to equivalent amount of free SSG (Figure 5B). PC-Chol on the other hand, masked the ability of entrapped SSG to bring down IL-10 (Figure 5C).

### Humoral response in PC-SA-SSG treated mice

**Figure 6:**
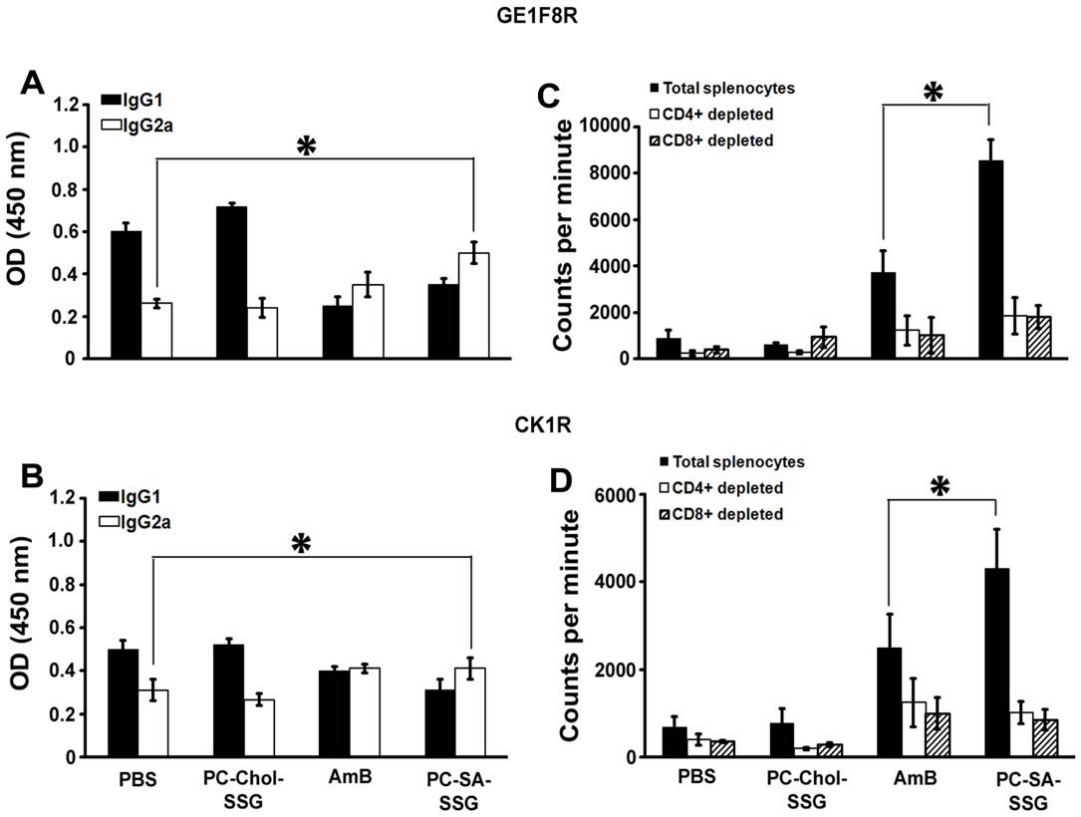
Humoral and antigen-specific proliferative response following treatment with PC-SA-SSG. (A, B) Sera from treated mice were analyzed individually by ELISA for detection of IgG1 and IgG2a antibodies in GE1F8R and CK1R-infected groups of mice (3-5 mice/group). The results are representative of two independent experiments and data represent mean ± SEM. * p<0.05 compared to PBS treatment. (C, D) Total, and CD4^+^ and CD8^+^ T cell-depleted splenocytes from treated groups were stimulated in vitro with *Leishmania* membrane antigen (LAg) (12.5* µ*g/ml). After 48 h, [^3^H] thymidine was added and cells were harvested subsequently. Proliferative index was measured as [^3^H] thymidine incorporation in counts per minute. Data represent mean ± SEM (3 mice/group) in triplicates. * p<0.05 compared to AmB therapy.

IgG2a levels are dependent on IFN-γ, whereas IgG1 levels correlate with IL-4. IgG2a and IgG1 are therefore used as surrogate markers for Th1 and Th2 responses [43]. PC-SA-SSG treatment mounted 2 and 1.3-fold (p<0.05) higher *Leishmania* membrane antigen (LAg)-specific IgG2a compared to respective GE1F8R, and CK1R-infected controls, and almost steady IgG1 levels concomitant with a steady secretion of IL-4. Similar results were also observed in mice treated with AmB, but not with PC-Chol-SSG treatment (Figure 6A, B).

### PC-SA-SSG-driven antigen-specific proliferative response

Impairment of cell-mediated immune response in active VL patients is reflected by marked T cell anergy specific to *Leishmania* antigens [31], [44] which is reversed by successful therapy. To investigate whether PC-SA-SSG can do so, we performed LAg-specific T cell proliferation assay. In contrast to PC-Chol-SSG therapy, PC-SA-SSG triggered 11 and 12-fold higher LAg-specific proliferation compared to respective GE1F8R and CK1R infected mice (Figure 6C, D), which were even higher than AmB treatment (p<0.05). The proliferative response was significantly blocked individually by both anti-CD4^+^ and anti-CD8^+^ antibodies inferring that marked proliferation was contributed by both subsets of T cells.

### Cyokine response and NO production

**Figure 7:**
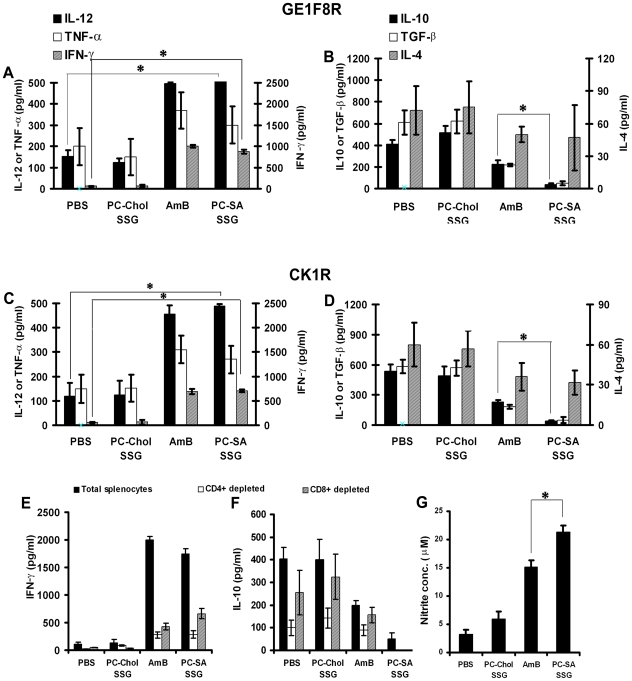
Differential pattern of cytokine and NO production following therapy with antileishmanial formulations in infected BALB/c mice. Splenocytes isolated from GE1F8R and CK1R-infected mice after indicated treatments were plated aseptically, and stimulated with LAg (12.5* µ*g/ml) for 72 h. (A, C) IFN-γ, IL-12, TNF-α and (B, D) IL-10, TGF-β, IL-4 cytokine levels in supernatants of splenocyte cultures were assayed by ELISA. Total, and CD4^+^ and CD8^+^ T cell-depleted splenocytes were stimulated as above and (E) IFN-γ and (F) IL-10 levels were measured after 72 h. Values represent the mean ± SEM (3–5 mice/group). (G) Leishmanicidal NO generation determined by Greiss assay method in supernatants of splenocytes derived from indicated groups. Data represent the mean ± SEM (3–5 mice/group). * p<0.05 compared to (A, C) PBS or (B, D,G) AmB therapy.

To compare the type of immunological response in *L. donovani*-infected mice with PC-SA-SSG liposome treated mice, detailed analysis of cytokine production was conducted in splenocytes of differently treated infected animals by ELISA at 12-wk post-infection. A general Th1 dominance in PC-SA-SSG treated mice was evident from 18 and 16-fold higher Interferon (IFN)-γ secretion from LAg-pulsed splenocytes, than respective GE1F8R and CK1R-infected mice (Figure 7A, C) (p<0.05) which was comparable with AmB therapy. In contrast, PC-Chol-SSG treated mice expressed no enhancement of IFN-γ. In vitro CD4^+^and CD8^+^ T cell depletion demonstrated significant role of the dichotomous T cell subsets in IFN-γ expression (Figure 7E). Elevated IFN-γ in PC-SA-SSG and AmB treated mice corresponded with a simultaneous upregulation of interleukin (IL)-12 and tumor necrosis factor (TNF)-α expression. IL-12 levels were elevated by 3.5 and 4-fold (p<0.05) and TNF-α levels showed 1.5 and 1.8-fold increase in PC-SA-SSG treated mice, compared to GE1F8R and CK1R-infected mice (Figure 7A, C).

We next investigated the altered expression of immunosuppressive cytokines of treated mice. Despite sustained levels of IL-4, PC-SA-SSG treated mice expressed negligible IL-10 (p<0.05) and transforming growth factor (TGF)-β compared to GE1F8R and CK1R-infected mice (Figure 7B, D). In vitro blocking demonstrated CD4^+^ cells as the main source of IL-10 (Figure 7F). In comparison AmB could only partially suppress the disease promoting cytokines, IL-10 and TGF-β highlighting the better curative response of PC-SA-SSG therapy. Failure to suppress IL-10 and TGF-β accounted for poor outcome of the therapy with PC-Chol-SSG (Figure 7B, D).

NO is the crucial killing effector molecule against leishmaniasis produced by IFN-γ-stimulated and NO synthase-induced classical macrophages. Th1 dominance in PC-SA-SSG treated mice correlated with 6-fold higher NO production than infected control, which was significantly higher than AmB treated group (Figure 7G) (p<0.05). The strong ability to suppress disease promoting IL-10 and to effectively trigger macrophage microbicidal molecule NO thus reversing the immunosuppressive condition towards Th1 type immune response, accounts for the radical cure elicited by PC-SA-SSG.

## Discussion

In this study we evaluated a new therapeutic approach with cationic liposomal SSG against SSG-resistant *L. donovani* parasites. Therapy with a single dose of SSG in PC-SA liposomes led to the successful cure of progressive SSG-resistant VL in BALB/c mice that was even better than AmB therapy in providing effective antileishmanial immunity and strong protection against reinfection. In contrast, equivalent amount of SSG in PC-Chol liposomes failed to evoke significant cure. Investigation into the probable mechanisms demonstrated that PC-SA-SSG could directly kill parasites irrespective of SSG-sensitivity owing to the leishmanicidal effect of PC-SA combined with higher accumulation of SSG within the amastigotes favored by this formulation, and a simultaneous protective immunomodulation of the host immune system.

To our knowledge this is the first demonstration of robust effective treatment against infection with SSG-resistant *Leishmania* parasites in mice with a liposomal SSG formulation. Previously reported anionic or neutral liposomal pentavalent antimonial formulations [19]–[25] were largely restricted to SSG-responsive strains alone, except one where differential organ dependent response was observed in primary infection model with SSG-resistant parasites [26]. As an extension to our previous observations on the therapy of PC-SA-SSG against SSG-sensitive *L. donovani* [29], we herein observed an equipotent effect of this therapy against non-healing infection with SSG-resistant parasites in BALB/c mice. Resistance mechanisms in clinical isolates differ from those active in laboratory generated strains [11]. PC-SA-SSG was equally effective against differentially originated GE1F8R and CK1R reflecting its strain independent antileishmanial activity. A single dose of 12 mg/kg of PC-SA-SSG was remarkably effective not only against liver but also splenic and bone marrow parasites which was even better than AmB therapy. There was a 10^5^-fold fall in viable parasites with less than 100 viable parasites observed in the organs after PC-SA-SSG therapy, which is a clear indication of nearly complete healing. In comparison, PC-Chol-SSG exhibited incompetence against SSG-resistant parasites. It is well known in VL that a successful therapy may not clear all the parasites from sites of infection but most T cell intact patients show long lasting clinical cure despite the presence of residual intracellular parasites [45]. Whereas PC-SA-SSG effectively controlled parasite visceralization on a secondary attack, AmB showed only a partial protection to reinfection, further strengthening the superiority of this formulation.

Prior experimental and clinical observations have pointed towards the importance of T cell mediated protective post-treatment mechanism in VL [45]. We tested the immunological outcome after chemotherapy in SSG-resistant *L. donovani*-infected mice treated with various therapies. Disease severity in BALB/c mice infected with SSG-resistant strains was associated with significantly hampered Ag-specific T cell proliferation, low expression of IL-12, TNF-α, IFN-γ and upregulation of suppressive cytokines IL-10 and TGF-β. Detailed immunological analysis of AmB and PC-SA-SSG treated mice showed enhanced T cell proliferation, persistent IgG1 levels, probably maintained by continued secretion of IL-4 [46], [47] along with increased IgG2a and upregulated IL-12 and IFN-γ production in LAg-pulsed splenocytes, which was lacking in PC-Chol-SSG treated group. Treatment with PC-SA-SSG also increased the level of another proinflammatory cytokine, TNF-α, which probably stimulates IL-12 driven IFN-γ secretion. Strong IL-12 driven IFN-γ and TNF-α triggering in PC-SA-SSG versus PC-Chol-SSG treatment suggests that these cytokines might be involved in the observed upregulated NO secretion for providing impressive levels of protection.

Although AmB induced significant Th1 responses, it failed to sufficiently suppress IL-10 and TGF-β production. On the other hand, PC-SA-SSG led to strong suppression of IL-10 and TGF-β production that correlated with successful resolution of infection. A growing body of literature correlates IL-10 and TGF-β with susceptibility to *Leishmania* infection [48]–[54], [42]. Since dominant host immunity over persistent infection was achieved by an ongoing Th1 response in the absence of immunosuppressive cytokines, animals treated with PC-SA-SSG could effectively prevent reinfection.

Such a profound leishmanicidal potential of SA-bearing SSG formulation against SSG-resistant parasites prompted us to investigate the probable mechanisms for this phenomenon. PC-SA-SSG treatment of infected macrophage cultures demonstrated a direct killing of parasites concomitant with a higher SSG accumulation inside the amastigotes. The leishmanicidal activity of the cationic PC-SA-SSG may be due to their preferential uptake by the macrophages [55] followed by their cytotoxic action in the parasitophorous vacuoles. Killing of parasites by PC-SA occurs through specific interactions of the liposomes with the parasite membrane phosphatidylserine (PS), leading to membrane disruption and depletion in cytosolic ATP levels [30]. The ATP-energized efflux transporters on parasite membranes responsible for the intracellular accumulation of drug [56] probably are rendered nonfunctional by PC-SA-SSG, enhancing SSG accumulation within resistant parasites. Additionally, liposomes are known to circumvent the action of membrane associated efflux pumps providing an alternate strategy to overcome drug resistance by increasing intracellular drug accumulation [32], [34]. There are also reports that SA-bearing liposomes are less leaky towards cationic drugs [57]. We used two dyes of different nature, Rh 123 which is entrapped in the lipid phase of the liposome and CF which is entrapped in the aqueous core. In addition, our aim behind using Rh 123 and CF was to assess their release profiles and see if PC-SA incorporation can increase their retention within resistant parasites, as the former is a well known Pgp substrate [34] and latter is known to be transported by proteins of the MRP transporter family [38]. Efflux of SSG in SSG-unresponsive parasites has been attributed to similar cellular processes [56]. Interestingly, PC-SA enhanced the intracellular retention of both the dyes, which are otherwise vigorously thrown out of the resistant cells [34], [58]. Although we didn't perform any experiments to confirm which efflux pumps is actually blocked, inhibited or downregulated in this case, but the retention studies clearly indicate a general decrease in efflux rates when liposomal incorporation was done. Thus, increased retention and slow release of the entrapped drug at the site of action add to the therapeutic advantage of PC-SA-SSG.

Cationic SA-bearing liposomes, in addition to having antileishmanial activity, greatly enhance the immunogenicity of associated antigens [40]. Antimonials can act effectively in the presence of a propicious immune response [59]. In view of our in vitro experiments on normal mice, it is clear that SSG exerted an inhibitory effect on IL-10 production that was augmented by incorporation in PC-SA liposomes. In vitro studies with only PC-SA also showed downregulation of IL-10 which was not the case with PC-Chol (data not shown). We have earlier observed successful downregulation of IL-10 and TGF-β along with an increase in IFN-γ levels in in vivo experiments on normal mice with only PC-SA [31], [60]. The exact mechanism for the IL-10 downregulation and effective generation of NO, however, remains unclear. SSG treatment is known to activate mitogen-activated protein kinase p38 (MAPK p38) and subsequently release TNF-α, which result in the production of NO in macrophages [61]. Recent reports suggest that resistant parasites modulate the host immunity to induce unresponsiveness to SSG therapy [62]. Moreover, SSG availability in resistant parasites is less, rendering the drug ineffective in the generation of host microbicidal molecules. As reported earlier [63], PC-SA liposomes may also activate the MAPK p38 pathway, in addition to increasing the intracellular retention of SSG. Any drug or drug combination activating the macrophage CD40 induced MAPK p38 pathway can also boost the IL-12 mediated antiparasitic function and ameliorate *Leishmania* infection by reinstating Th1 response [64]-[66]. At the doses used, it is possible that PC-SA, along with SSG induces a MAPK mediated signalling pathway, downregulating the pro-parasitic IL-10 secretion, ultimately leading to killing of the parasite and restoration of the protective immunity.

Promising therapeutic effect and protection against SSG-unresponsive *L. donovani* infection can be attributed to multiple driving forces exerted by the combined activity of SSG and PC-SA leading to enhanced accumulation of SSG within parasites, direct killing of parasites induced by PC-SA and the switch of immunosuppressive humoral and cell-mediated responses to a protective Th1 type. This synergistic approach gains importance in today's scenario where it can help save some of the potent drugs against infectious diseases which are facing extinction due to emergence of resistance, since resistance to a combination therapy is less likely to occur.
